# Circular RNA HIPK3 is a Prognostic and Clinicopathological Predictor in Malignant Tumor Patients

**DOI:** 10.7150/jca.40001

**Published:** 2020-04-27

**Authors:** Gao Wenzhe, Xu Jiahao, Peng Cheng, Zhu Hongwei, Yu Xiao

**Affiliations:** 1 Department of Hepatobiliary and Pancreatic Surgery, The Third Xiangya Hospital, Central South University, Changsha, Hunan Province, China 410013; 2 Medical College of Xiangya, Central South University, Changsha, Hunan Province, China 410013; 3 Department of Gastroenterology, The Third Xiangya Hospital, Central South University, Changsha, Hunan Province, China 410013

**Keywords:** HIPK3, circRNA, cancer, prognosis, meta-analysis

## Abstract

**Objectives**: Circular RNA Homeodomain Interacting Protein Kinase 3 (circHIPK3) is one of the most researched circRNAs in recent 5 years. Many individual studies confirmed that aberrantly expression of circHIPK3 held prognostic value in various tumors. Thus, the aim of this meta-analysis is to assess its prognostic potentials and functions in malignant tumors.

**Materials and methods**: Multiple databases were carefully searched for articles published about circHIPK3 over the past 10 years. Hazard ratios (HRs) or odds ratios (ORs) with 95% confidence intervals (95% CIs) were calculated to demonstrate prognostic value of circHIPK3 using Stata 15.0 software.

**Results**: 8 studies including 1175 patients were ultimately enrolled in this meta-analysis. Pooled results showed that abnormal expression of circHIPK3 was significantly correlated with unfavorable OS (pooled HR=2.12, 95% CI: 1.69-2.66) and DFS/PFS (HR=2.28, 95% CI: 1.67-3.10) in cancer patients. Additionally, abnormal expression of circHIPK3 was also related to the distal metastasis of the tumors (OR: 3.27, 95%CI: 2.16-4.97, p<0.001).

**Conclusions**: Abnormal expression of circHIPK3, no matter high or low expression, was associated with poor clinical outcomes in multiple cancer types. More comprehensive studies were required to verify and strengthen the clinical value of circHIPK3 in human malignant diseases.

## Introduction

Circular RNAs (circRNAs) represent an emerging class of endogenous non-coding RNA which has become a new research hotspot since 2012. They share a closed loop structure and are abundantly enriched in eukaryotes 1. Most of circRNAs contain exon sequences from their original genes and are highly conserved in different species. Besides, they often show expressional specificity in different tissues and developmental stages 1. With the development of high-throughput deep RNA sequencing technology and bioinformatic methods, a new correlation has been established between circRNAs and many pathological processes, especially malignant neoplastic diseases, including cancer and sarcoma 2. Researches in recent years have proved that circRNAs play a pivotal role in the development and progression of multiple cancers. Functions include (1) micro-RNA sponges or protein binding in cytoplasm; (2) transcriptional regulators in nucleus; (3) RNA alternative splicing modulators in nucleus 3-4. Furthermore, circRNAs exhibit a more stable nature than linear RNAs and are insensitive to the degradation of nucleases 5. All these characteristics may provide an extra potential for developing new clinical diagnostic and prognostic markers for cancer patients.

Circular RNA Homeodomain Interacting Protein Kinase 3 (circHIPK3 or circ_0000284) was first discovered in 2013 by Jeck, WR et al 6. In 2016, Zheng, Q et al published the first study about the correlation between circHIPK3 and tumors. They proved that circHIPK3 functioned as a regulator of cancer cell growth by sponging multiple microRNAs 7. Since then, many researchers have explored the specific relationship between circHIPK3 and various malignant diseases. However, controversies emerged. In some kinds of tumors, high expression of circHIKP3 showed an oncogenic effect, while in other tumors it was fully opposite. Most researches have focused on the tumor promoting function of circHIPK3. For example, Chen et al 8 proved that the loss of circHIPK3 induced protective autophagy via MIR124-3p-STAT3-PRKAA/AMPKa axis in STK11 mutant lung cancer cell lines (A549 and H838). Li et al 9 found the role of circHIPK3 in inducing cell proliferation and inhibiting apoptosis in NSCLC through miR-149. Additionally, Chen et al 10 showed that circHIPK3 promoted cell proliferation and migration by sponging miR-124 and further downregulating aquaporin 3 (AQP3) expression. Nevertheless, there were also a few articles elucidating the low expression level and anti-tumor effect of circHIPK3. Li et al 11 found a very low expression level of circHIPK3 in bladder cancer (BC) samples compared with that in normal tissues; over-expression of circHIPK3 could inhibit migration, invasion and angiogenesis of BC cell lines in vitro by abundantly sponging miR-558 and further suppressing the expression of heparanase (HPSE). Similarly, Ma et al 12 demonstrated that circHIPK3 overexpression significantly suppressed proliferation, migration and invasion of osteosarcoma cells.

No matter high or low expression in tumors compared with normal tissues, it has become an indisputable fact that circHIPK3 could affect the development and progression of malignant diseases. As one of the most studied circRNAs, we proposed a new question: did circHIPK3 possess the value of prognostic and clinicopathological significance in cancer patients? To solve this problem, we designed the following systematic review and meta-analysis. As far as we have learned, this is the first research so far to integrate all available literatures to determine the clinical value of a certain circRNA.

## Material and Methods

### Searching Strategy

Two reviewers (Gao WZ and Zhu HW) independently performed the literature retrieval in July 2019. The following databases were searched: PubMed, PMC, EMBASE, Web of Science, Cochrane Library, China National Knowledge Infrastructure (CNKI), and Wanfang Database. The time range of search was from July 2010 to July 2019 (the latest 10 years). The following keywords were used for a complete searching of all the literatures related to circHIPK3: “circHIPK3” OR “circRNA HIPK3” OR “circular RNA HIPK3” OR “circ_0000284” OR “circular RNA Homeodomain Interacting Protein Kinase 3” OR “circRNA Homeodomain Interacting Protein Kinase 3” OR “HIPK3” OR “Homeodomain Interacting Protein Kinase 3”.

### Inclusion and Exclusion Criteria

Inclusion criteria: 1) The expression of circHIPK3 was detected in any neoplastic disease of human; 2) Correlation between circHIPK3 and prognosis was reported; 3) Hazard ratio (HR) and the corelated p-value OR 95% CI were provided, or sufficient data was available for calculating HR with 95% CI; 4) Patients were stratified according to the expression level of circRNA HIPK3. Literatures which could not meet all of the points above were excluded.

### Data Extraction and Quality Assessment

Two investigators (Gao WZ and ZHU HW) independently extracted data from identified studies according to unified form. Extracted data elements included the following records: 1) Name of the first author, publication year, country of origin, study design, cancer type, abnormal expression of circHIPK3 in cancer, sample size, expression pattern, tumor stage, definition of high and low expression groups, method of detecting expression, follow-up time, outcome measures; 2) Hazard ratio (HR) with 95% CI for overall survival (OS) or other survival-related prognostic indicators; 3) Case numbers for high expression and low expression, lymph node metastasis (LNM), distant metastasis (DM), lower differentiation (LD), and tumor-node-metastasis (TNM) stage.

### Statistical Analysis

Stata version 15.0 (Stata Corporation, College Station, TX, USA) was used to calculate all statistical indicators in this meta-analysis. Higgins I^2^ statistics and Cochran's Q-test were applied to assess the heterogeneity among studies. When the percentage of I^2^ was greater than 50% or P_het_ less than 0.05, a random-effects model was used, otherwise the fixed-effects model was applied. Begg's and Egger's test were utilized to detect the publication bias. Although only 8 researches were included in this study, sensitivity analysis was still performed by omitting the study one by one to assess the effects on the pooled results as supporting evidence to further detect heterogeneity. A p-value < 0.05 was considered statistically significant.

## Results

### Study selection and Characteristics

**Figure 1:**
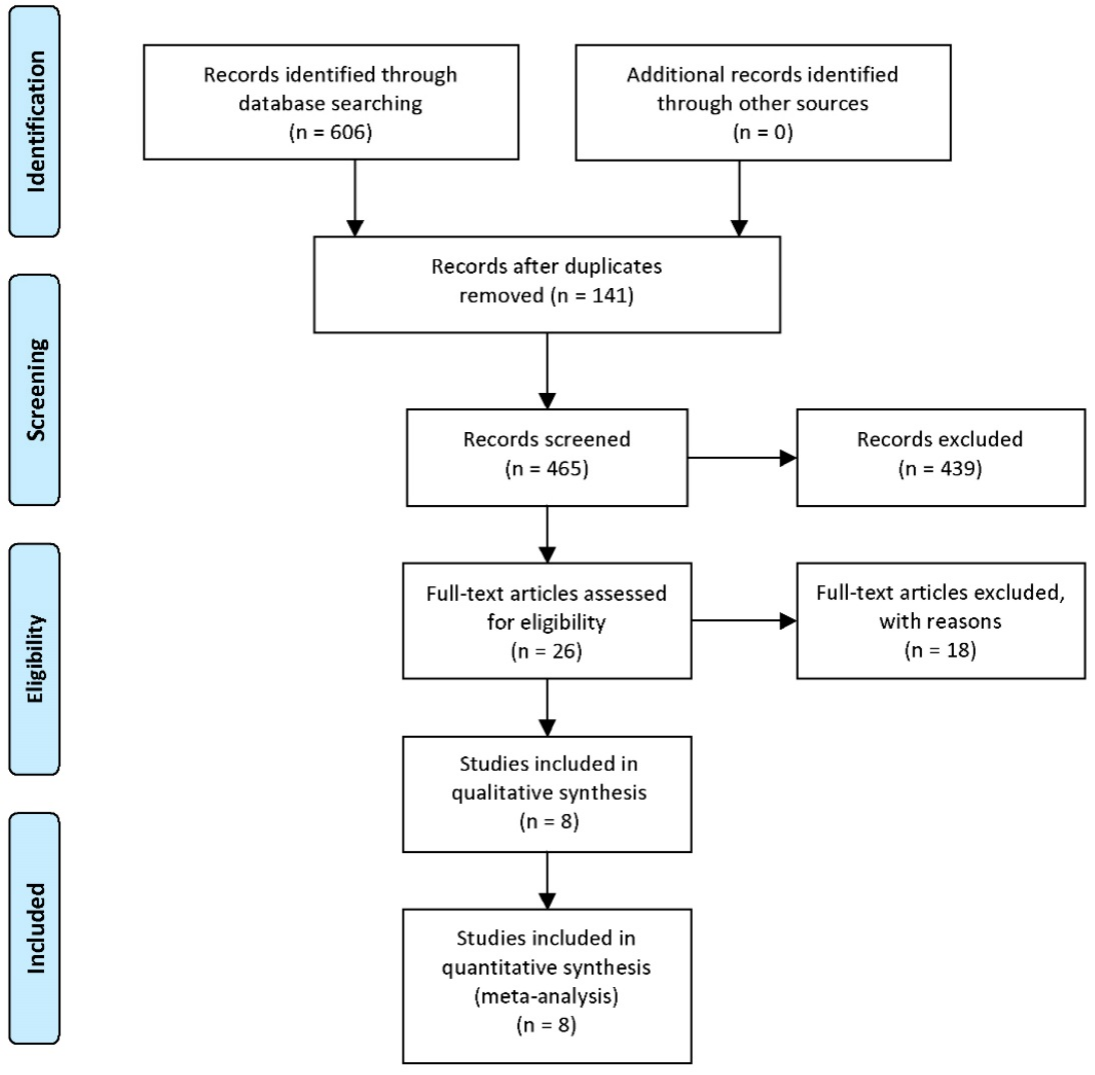
Steps for screening eligible articles.

A total of 465 papers were retrieved from the databases mentioned above after duplications removed. Titles and abstracts were then carefully overviewed. After that, 26 articles were left as 439 articles were excluded for the following reasons: reviews or conference reports, animal and/or in vitro researches with no patients involved, only HIPK3 mRNA expression mentioned but no circRNA, human tissues included but clinical outcomes not mentioned. After further screening of the remaining 26 studies, 18 were excluded due to survival data missing or difficult to extract. Finally, 8 available studies were considered applicable to this meta-analysis. The detailed steps for screening eligible articles are presented in **Figure 1.**

The eight incorporated studies were published from 2017 to 2019, this time period was in line with the advancement of detection techniques and the research boom of circRNAs. These studies were all identified as high-quality judged by Newcastle-Ottawa Scale (NOS) >= 7 points. A total of 1175 cancer patients were enrolled, with a mean subject size of 146.9 ranging from 63 to 457. Seven studies measured the expression of circHIPK3 by quantitative real-time polymerase chain reaction (qRT-PCR), another one study used RNA-Seq as their detection method; the expression of circHIPK3 in cancer tissues was measured in 6 studies and 2 studies detected it in patients' serum (one study for chronic myeloid leukemia (CML), which was a typical hematological malignancy, another one for nasopharyngeal carcinoma). They all reported the relationship between the expression of circHIPK3 and the prognostic value of patients.

Among all eight studies, seven reported patients' OS, two focused on DFS, another one focused on PFS, respectively. Six studies reported the relationship between circHIPK3 and DM, four studies estimated tumor differentiation and another three and four focused on LNM and TNM, respectively. Cancer types in these studies consisted of epithelial ovarian cancer (EOC), nasopharyngeal carcinoma (NPC), bladder cancer (BC), colorectal cancer (CRC), lung cancer (LC) and chronic myeloid leukemia (CML). All included studies were retrospectively researched. They were conducted in different countries, but mainly in China (6 in China and 1 in Denmark). **Table 1** and** Table 2** summarized the main information and data of all included studies.

Although the excluded studies lacked the data necessary for meta-analysis, we still tried our best to extract information regarding the clinical correlation of circHIPK3 or the function of circHIPK3 in different cancer types based on the conclusions from their in vitro or in vivo data. After a further screening, another 6 studies were presented in **Table 3**.

## Results of the Meta-analysis

### Relationship between the expression of circHIPK3 and patient prognosis

**Figure 2:**
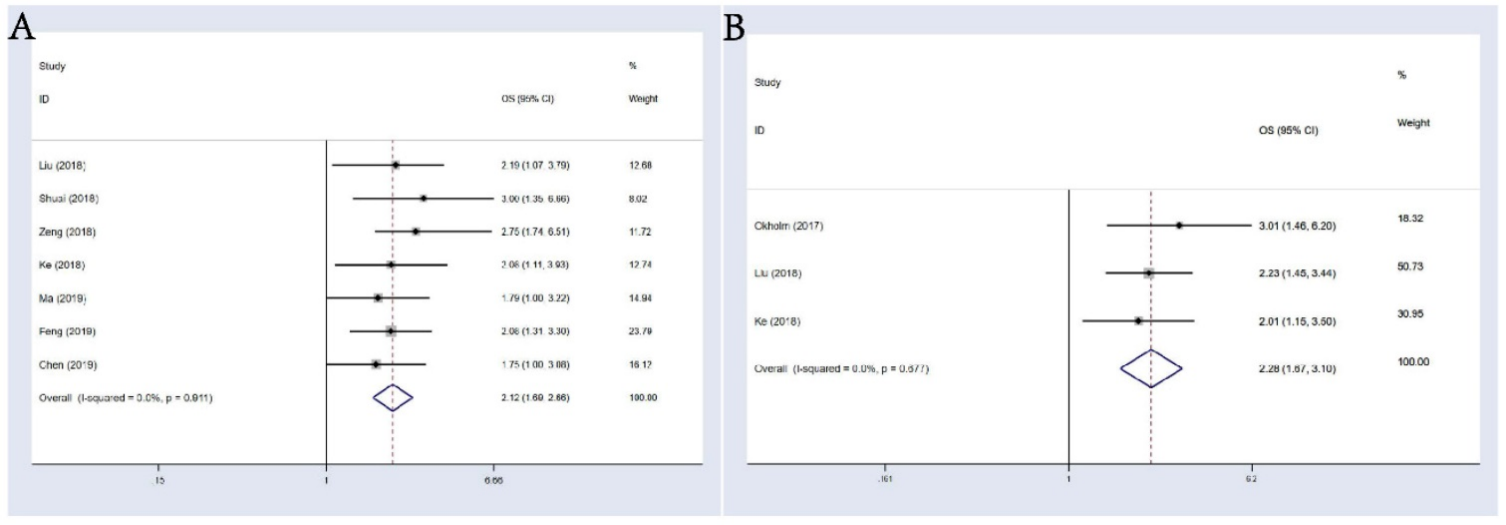
Meta-analysis of the pooled HRs of OS. **(A)** and DFS&PFS; **(B)** of patients with abnormal (high or low) circHIPK3 expression.

7 studies composed of 718 cancer patients were conducted to analyze OS; a fixed-effects model was adopted (I-squared=0.0%, p=0.911). In the result of pooled analysis, we noticed an obvious correlation between the differential expression of circHIPK3 and poorer OS in cancer patients (pooled HR=2.12, 95% CI: 1.69-2.66; **Figure 2A**). Moreover, a total of 3 studies including 589 patients investigated the prognostic value of circHIPK3 on cancer progression or recurrence, in which a progression-free survival (PFS) or disease-free survival (DFS) was calculated. We also found a pooled HR of 2.28 (95% CI: 1.67-3.10; **Figure 2B**) in a fixed-effects model (I-squared=0.0%, p=0.677).

**Figure 3:**
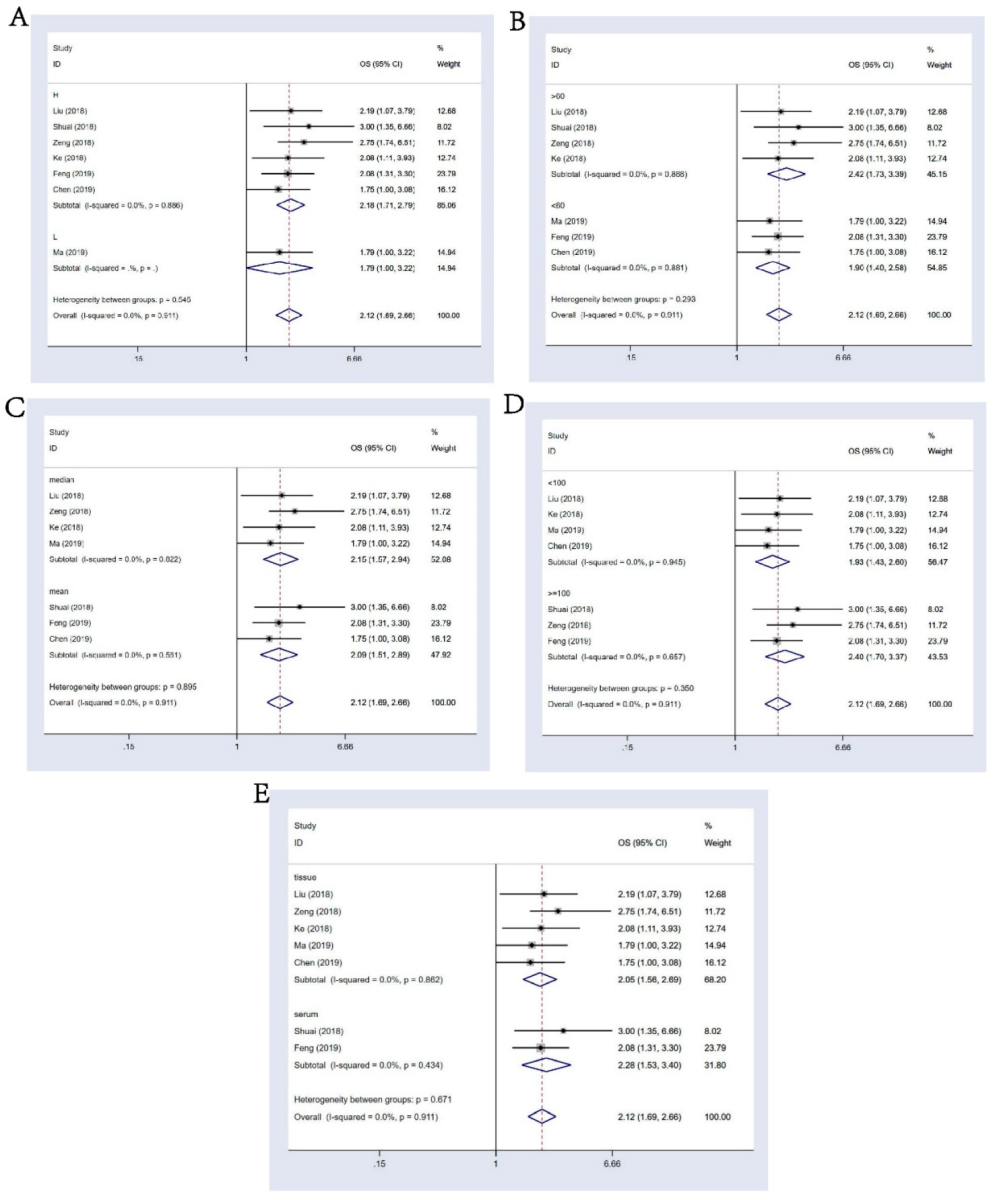
Subgroup analysis for OS. **(A)**: stratified by circHIPK3 expression status; **(B)**: by follow-up time; **(C)**: by criterion of circHIPK3 high expression (HE)** (D)**: by sample size; (E): by sample type

In the result of subgroup analysis by the expression status of circHIPK3, we found circHIPK3 could act as a prognostic factor in the group that had a higher expression of circHIPK3 in tumors than in normal tissues (HR: 2.18, 95% CI: 1.71-2.79; **Figure 3A**); Since only one document reported OS data for tumors with lower expression of circHIPK3 (Ma, 2019 for osteosarcoma), we could not draw a conclusion for this group. Other stratification analysis for all subgroups were presented in detail in **Figure 3B-3E**, we discussed the prognostic value of circHIPK3 in groups of follow up time(>60 or <=60 months, p for heterogeneity = 0.293 ), criterion of “high-expression”(based on the median or the mean level, p for heterogeneity = 0.895), sample size (>=100 or <100 patients, p for heterogeneity = 0.350) and sample type (tissue or serum, p for heterogeneity = 0.671). It turned out that differential expression of circHIPK3 is related to the worse OS in every single subgroup and these subgroups had no significant impact on the output of the overall survival.

### circHIPK3 Expression and Clinicopathological Characteristics

**Figure 4:**
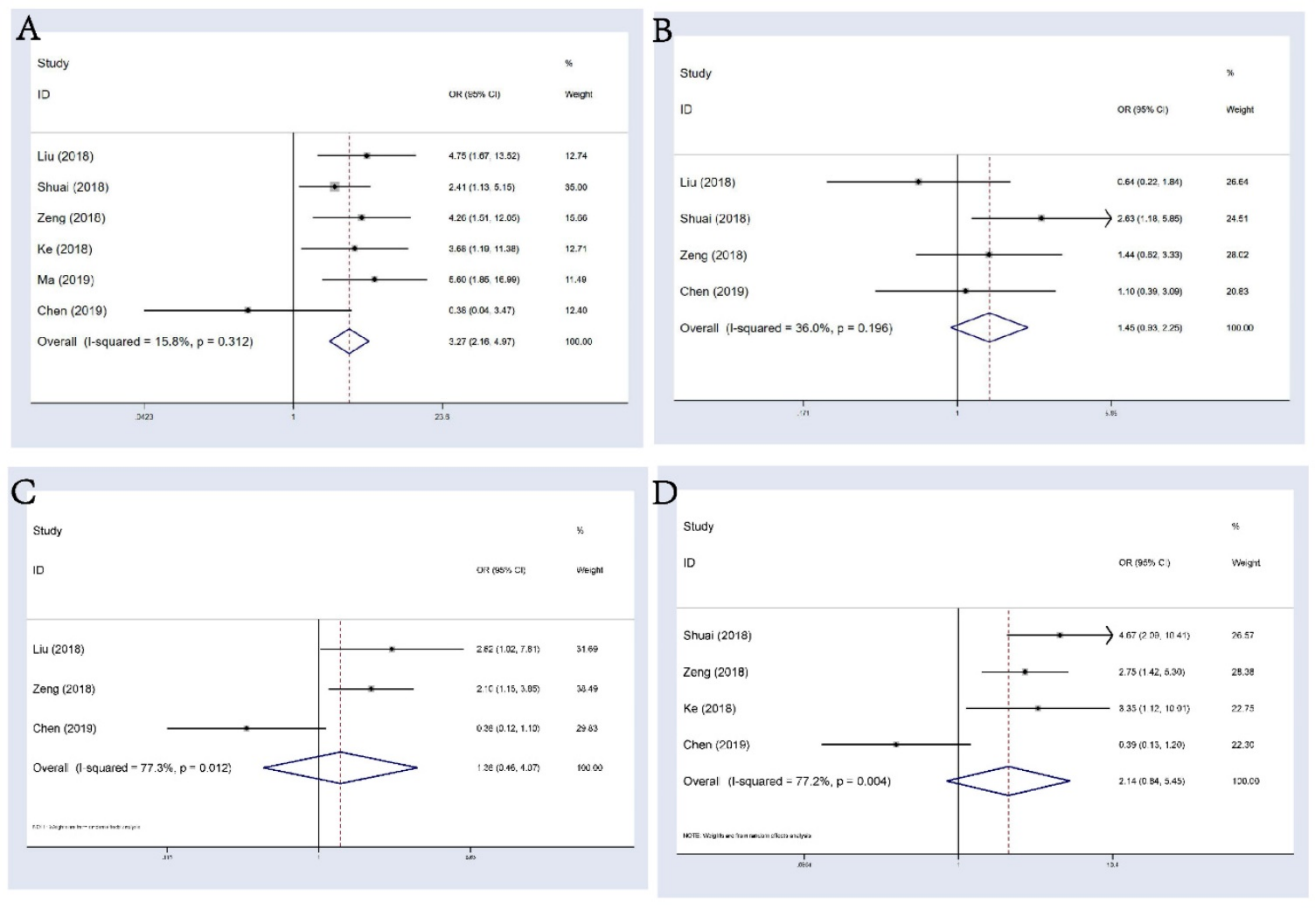
Forest plots of combined analyses associated with circHIPK3 expression. **(A)**: DM; **(B)**: differentiation; **(C)**: LNM; **(D)**: TNM stage.

6 studies consisting of 555 patients reported the correlation between circHIPK3 and distant metastasis (DM) in multiple tumors. A fixed-effects model was applied (I^2^=15.8%, P_het_=0.312), the pooled results showed that patients with abnormal circHIPK3 expression had a significant higher risk of DM (OR: 3.27, 95%CI: 2.16-4.97, p<0.001; **Figure 4A**). However, evidence was still not enough to say that abnormal expression of circHIPK3 was correlated with tumor differentiation, lymph node metastasis (LNM) or TNM stage (**Figure 4B-D**), although trend was seen that abnormal circHIPK3 expression might be related to these undesirable clinicopathological factors. We noticed that Chen's study always showed an opposite tendency to other studies in these analyses and the ORs for this study were always statistically insignificant. Since this study found that high expression of circHIPK3 is corelated with a poorer OS in lung cancer patients, we believed that this weird tendency should be attributed to a low case number included in this research. There were only 69 patients included and only 25 patients were defined as “high expression”. Thus, the incidence of every event in this study was low and likely to be unstable, leading to the occurrence of this small accident.

### Publication Bias

**Figure 5:**
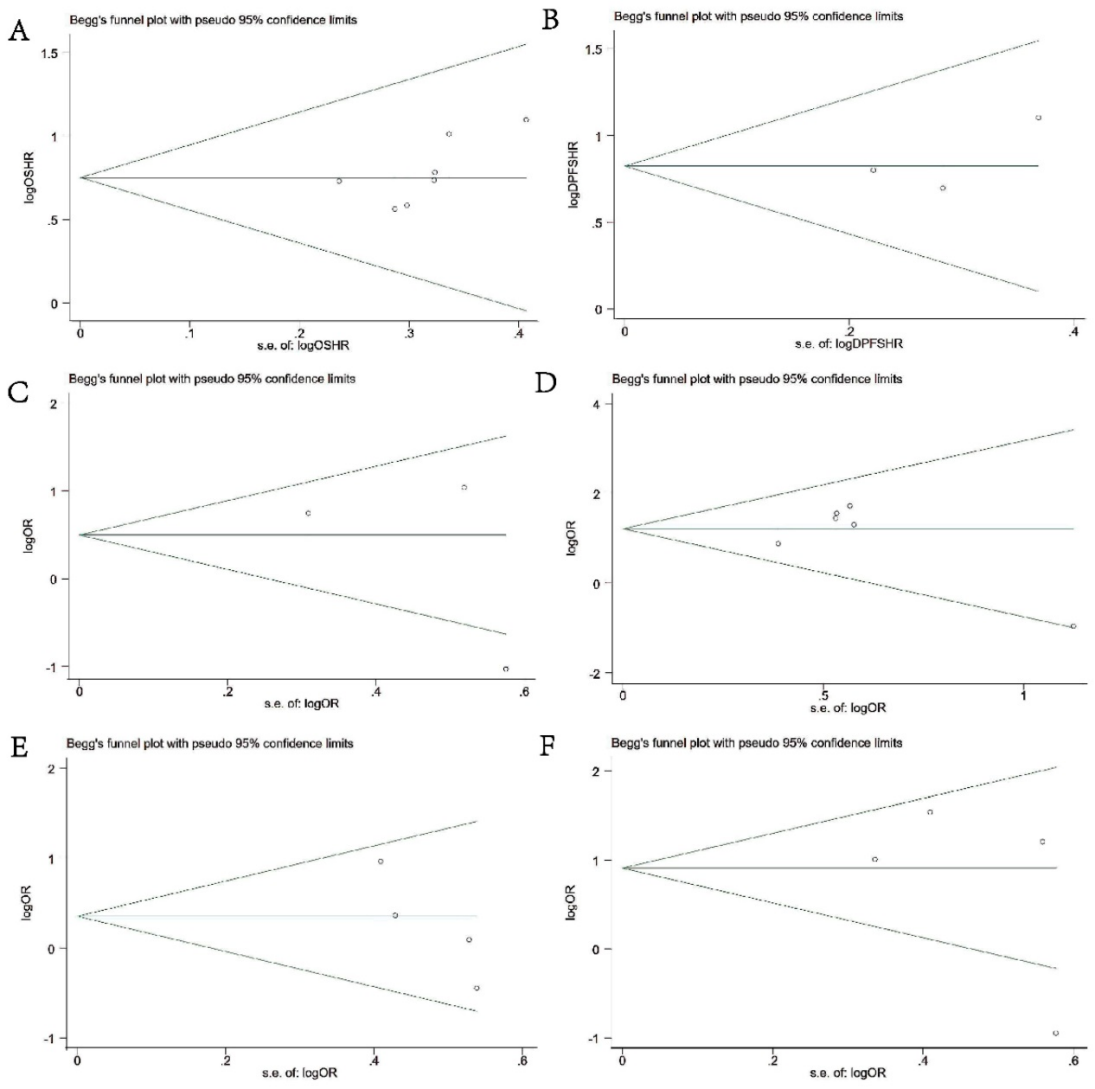
Begg's funnel plots of publication bias test. **(A)** OS; **(B)** DFS or PFS; **(C)** DM; **(D)** differentiation; **(E)** LNM; **(F)** TNM stage.

Begg's & Egger's test and funnel plot were used to evaluate the publication bias in this meta-analysis. As shown in **Figures 5A-5F**, the Begg's funnel plots with pseudo 95% CIs were symmetric. The test results also indicated no significant publication bias in this meta-analysis.

### Sensitivity Analysis

**Figure 6:**
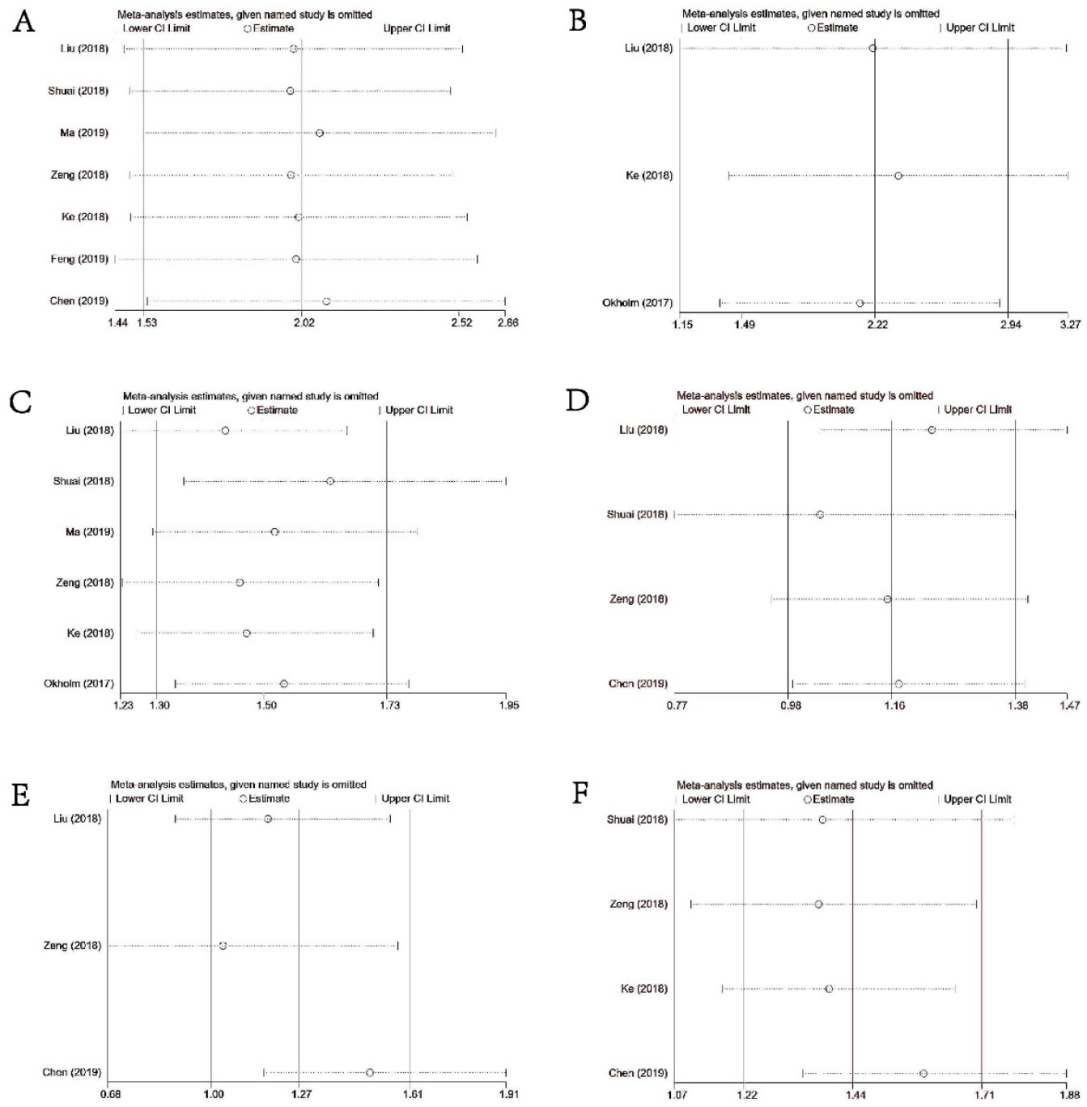
Sensitivity analysis under specific model. **(A)**: for OS; **(B)**: for DFS or PFS; **(C)**: for DM; **(D)**: for differentiation; **(E)** for LNM; **(F)** for TNM stage.

As illustrated in **Figures 6A-6F**, sensitivity analysis suggested that our results were comparatively credible and stable.

## Discussion

Circular RNA HIPK3 was first identified in 2013, when JECK WR et al. 6 found that *HIPK3*, the gene of a paralogous kinase, could produce abundant circRNAs from its second exon in both human and mice through RNA-seq; moreover, they found circHIPK3 contained an AUG translation start but could not bound to ribosome, so there was no ability for circHIPK3 to encode proteins. As one of the earliest identified tumor-related circRNAs, circHIPK3 has been proved to play carcinogenic roles in many kinds of tumors. Overexpression of circHIPK3 promoted the progression of many cancers. In addition to the literature we listed in the **Introduction** section, Chen D 20 and Cai C 23 independently found circHIPK3 was overexpressed in cell lines and tissues of prostate cancer. It functioned as a sponge to absorb miR-338-3p and miR-193a-3p and then promoted proliferation and invasion. There were many other examples like this, including most studies in our meta-analysis (6 of 8). However, what could not be ignored was that circHIPK3 low expression also exerted a carcinogenic effect in several kinds of tumors, which meant that circHIPK3 could also act as a tumor suppressor. Among the included studies, Ma 12 and Okholm 15 separately proved that circHIPK3 was low expressed in osteosarcoma and bladder cancer, this downregulation was also related to the progression of tumors. Low expression of circHIPK3 in bladder cancer and its accompanying cancer-promoting effect were also proved by Li et al 11 as mentioned in the **Introduction** part. Considering that circHIPK3 could sponge a variety of different miRNAs that might have either tumor promoting or suppressing function, it was not surprising that circHIPK3 exhibited a dual effect in different tumors. However, how was the expression of circHIPK3 regulated in different tumors? In addition to the sponge function, what were the other roles that circHIPK3 might play in the development of these diseases? Further researches were still needed to answer these questions.

To our knowledge, this was the first meta-analysis discussing the prognostic value of a certain circRNA in all human neoplastic diseases. We demonstrated that the abnormally differential expression of circHIPK3 had a significant correlation with clinical outcomes associated with poor prognosis. The higher the degree of differential expression of circHIPK3 in tumors, the worse the OS for cancer patients (HR=2.12, 95% CI: 1.69-2.66). Furthermore, circHIPK3 was also related to the disease-free survival (DFS) or progression-free survival (PFS) of patients, with a pooled HR=2.28 (95% CI: 1.67-3.10), although the number of papers included was relatively small (only 3 studies). In addition, the differential expression of circHIPK3 was also concerned with distal metastasis (DM) of tumors (OR: 3.27, 95%CI: 2.16-4.97). Results from the sensitivity analysis and publication bias evaluation further indicated that our analysis was credible and stable.

Although this meta-analysis was conducted with standard procedure and rigorous statistics, limitations still existed and should be emphasized. First, a relatively small number of patients and studies included could not be ignored; during the process of our searching and reading, we found some contradictory points that possibly caused by this problem. For instance, in the research from Liu N et al. 13, circHIPK3 was up-regulated and predicted worse prognosis in epithelial ovarian cancer (EOC) tissues and cells compared with adjacent normal tissues and ovarian epithelium cell lines; higher circHIPK3 level was an independent predictor of poor prognosis in EOC patients. However, Fang T et al 24 published that circHIPK3 was highly expressed in normal ovarian tissue cells compared with that in all the ovarian cancer cell lines and exhibited a tumor-suppressive function. This contradiction was not reflected in this meta-analysis, because no survival data was available in the latter study. Such a contradiction was difficult to explain, different PCR primer designs and cell lines used for research might be the cause of it, which showed that so far there were still irregularities in the research of circRNAs. Second, the quantity of included studies was not sufficient enough for a more comprehensive analysis for other prognostic indicators and clinic-pathological features. Tendency could be noticed on tumor differentiation, TNM staging and lymph node metastasis, but limited by the number of patients included, we were unable to draw statistically significant conclusions. Third, given that all included studies were retrospective, issues like heterogeneity and publication bias might be unavoidable. Fourth, patients included were mainly Chinese population, which might affect our conclusions in a wider range of applications. Finally, most of our survival data was extracted from the K-M survival curve in the article and our estimated HRs might lack accuracy due to the lack of accurate information of patient survival and censored data. Since circular RNAs are currently one of the most popular research areas, we highly recommend scientists and clinicians to further explore their mechanism of affecting tumorigenesis and tumor progression and the impact on patients' clinical outcomes.

To put in a nutshell, this meta-analysis highlighted the clinical effect of circHIPK3 in predicting patients' survival and distal metastasis in multiple cancers. Higher quality studies with larger sample size were required for a more complete confirmation for other roles of circHIPK3 expression in human cancers.

## Figures and Tables

**Table 1:** Main characteristics of all studies included in the meta-analysis.

First author, year	Country	Cancer type	Study design	Sample size	HE (%)	Stage	Cutoff value	Follow-up years	circHIPK3 assay	Detected sample	Outcome measures	NOS score	

Chen,2019 8	China	LC	R	76	32.89%	TNM I-IV	mean	<=60 months	RT-qPCR	Tissue	OS	9	
Ma, 2018 12	China	Osteosarcoma	R	82	45.12%	Enneking I-III	mean	<=60 months	RT-qPCR	Tissue	OS	8	
LIU,2018 13	China	EOC	R	69	50.72%	FIGO I-III	median	>60 months	RT‑qPCR	Tissue	OS, DFS	8	
SHUAI,2018 14	China	NPC	R	150	70.00%	TNM I-IV	mean	>60 months	RT‑qPCR	Serum	OS	7	
Okholm,2017 15	Denmark	BC	R	457	49.89%	TNM Ta-T1	median	>60 months	RNA-Seq	Tissue	PFS	8	
Zeng,2018 16	China	CRC	R	178	50.00%	TNM T1-T4	median	>60 months	RT-qPCR	Tissue	OS	8	
Ke, 2018 17	China	NPC	R	63	50.79%	TNM T1-T4	median	>60 months	RT-qPCR	Tissue	OS, DFS	7	
Feng,2019 18	China	CML	R	100	62.00%	NM	mean	<=60 months	RT-qPCR	Serum	OS	9	

**Abbreviations and Comments: EOC**, epithelial ovarian cancer; **NPC**, nasopharyngeal carcinoma; **BC,** bladder cancer; **CRC**, colorectal cancer; **CML**, chronic myeloid leukemial **LC**, lung cancer; **R,** retrospective; **HE**, high expression; **Cutoff value** means the criterion of HE in every certain research that is based on whether the mean or median expression level of circHIPK3; **OS**, over survival; **DFS**, disease-free survival; **PFS**, progression‐free survival; **qRT-PCR**, quantitative real-time polymerase chain reaction; **RNA-Seq**, RNA Sequencing

**Table 2:** HRs and 95% CIs of cancer prognosis and progression associated with circHIPK3 expression in all included studies.

First author, Year	Country	Cancer type	Abnormal expression status	Case number of circHIPK3 expression (percentage%)										HR(95%CI) for OS	HR(95%CI) for DFS/PFS
				High	Low	High with LNM	Low with LNM	high with DM	low with DM	high with poor Diff	low with poor Diff	high with TMN III-IV	low with TMN III-IV		
Chen, 2019	China	LC	high	25	51	5 (20.0)	21 (41.2)	1 (4.0)	5 (9.8)	8 (32.0)	15 (29.4)	5 (20.0)	20 (39.2)	* 1.755 (1.000-3.081)	NM
Ma, 2019	China	osteosarcoma	low	37	45	NM	NM	5 (13.5)	21 (46.7)	NM	NM	NM	NM	*1.794 (1.000-3.218)	NM
LIU, 2018	China	EOC	high	35	34	16 (44.4)	9 (27.3)	19 (52.8)	8 (24.2)	8 (22.2)	12 (36.4)	NM	NM	#2.188 (1.065-3.788)	#2.226 (1.445-3.444)
SHUAI, 2018	China	NPC	high	105	45	NM	NM	47 (44.8)	13 (28.9)	45 (42.9)	10 (9.5)	60 (57.1)	10 (22.2)	*2.998 (1.350-6.658)	NM
Okholm,2017	Denmark	BC	low	228	229	NM	NM	NM	NM	NM	NM	NM	NM	NM	*3.010 (1.461-6.199)
Zeng, 2018	China	CRC	high	89	89	46 (51.7)	30 (33.7)	18 (20.2)	5 (5.6)	15 (16.8)	11 (12.4)	38 (42.7)	19 (21.3)	#2.750 (1.740-6.510)	NM
Ke,2018	China	NPC	high	32	31	NM	NM	15 (46.9)	6 (19.4)	NM	NM	25 (78.1)	16 (51.6)	*2.084 (1.107-3.926)	*2.008 (1.152-3.502)
Feng, 2019	China	CML	high	62	38	NM	NM	NM	NM	NM	NM	NM	NM	*2.075(1.306-3.298)	NM

**Abbreviations and Comments: Abnormal expression status** means the reported expression of circHIPK3 in tumor tissue/serum compared with normal tissue/serum samples. **Case number of circHIPK3 expression** means the number of cases that were defined as “high or low expression of circHIPK3” according to the criterion of every single research (based on median or mean expression level of circHIPK3). **NM**, not mentioned; **LNM**, lymph nodes metastasis; **DM,** distant metastasis; **poor Diff**, poor differentiation; *HR calculated from K-M survival curves; **#**HR directly from multivariate analysis.

**Table 3:** Further information extraction from studies that were not eligible to be included in the meta-analysis

First author, year	Country	Cancer type	Detected sample	Abnormal expression status	Related Clinicopathological Factors
Chen, 2018 10	China	HCC	Cell lines in vitro	High	Promoting cancer proliferation and migration
Li, 2017 11	China	BC	Patients' tissue	Low	Negatively correlated with tumor grade, invasion and lymph node metastasis
Cheng, 2018 19	China	GC	Patients' tissue	High	Positive corelated with invasion
Dong, 2019 20	China	PC	Cell lines in vitro	High	Promoting cancer proliferation and invasion
Jin, 2018 21	China	glioma	Cell lines in vitro	High	Promoting cancer proliferation and migration
Kai, 2018 22	China	GBC	Cell lines in vitro	High	Promoting cancer cell growth and inhibiting apoptosis

**Abbreviations and Comments: Abnormal expression status** means the reported expression of circHIPK3 in tumor tissue/cells compared with normal samples.** GC**, gastric cancer;** BC**, bladder cancer; **PC**, prostate cancer**; HCC**, hepatocellular carcinoma;** GBC**, gallbladder cancer;
